# Supplementary material for: Confirmatory factor analysis and exploratory structural equation modelling of the factor structure of the Depression Anxiety and Stress Scales-21
Source: PLoS One. 2020 Jun 5;15(6):e0233998. doi: 10.1371/journal.pone.0233998 (PMC7274426; doi:10.1371/journal.pone.0233998)
Supplement: Attachment — Submitted filename: revision_PLOSone.docx [file pone.0233998.s002.docx]

The subject is of interest and the manuscript has been performed with quality methodological. However, some aspects should be worked on, namely the quality of the writing and the content to be expressed in each part of the manuscript.

Introduction

- The authors don't have provided literature to justify the need for testing the DASS-21. The beginning "The DASS-21 has been derived from...." is not an appropriate way to start a manuscript. It should include a brief introduction of the topic to be addressed, of the importance of this scale... And not start with a psychometric approach. In this same line, concepts such as CFA and ESSEM are first introduced to be explained in the final part of the introduction; this does not facilitate the understanding of the subject.

- The authors make multiple statements throughout the translation that should be referenced, giving rise to confusion because they seem to have given it.

- The introduction is excessively long and confusing in its presentation. It needs to be worked on in-depth. It is not properly structured, with ideas repeated throughout the text that do not facilitate understanding and a vision of progression and organization.

- Acronyms must be specified the first time they appear. Even the name of a scale.

- On page 6, refer to "minor misspecifications", please clarify such general expressions.

Method:

- There is no need to indicate age by gender.

- Better express the statistical results (M = , SD = )

- The expression (t[736]=0.05, ns), is incorrectly expressed. Please include the statistic t correctly and the p.

- It is unnecessary to include the descriptive for the DASS items.

- It would be convenient to include other types of sociodemographics in the table, such as the year, the gender, the nationality, socioeconomics level.

- The last paragraph of the participants is for discussion, not methods.

- More detailed information needs to be provided on sample and recruitment, procedure.

- It would be recommended to reduce the analysis data section.

- It has not been included which type of statistical tests have been carried out in the validity of the tool.

Results

- Summarize the results in the text if they are set out in the tables.

- It is not necessary to explain in detail in the text the factorial loads of each of the items. Summarize and specify the different and/or relevant results. Also, organize it in a detailed way by the factor in each of the models. As it is now, it is confusing and disorganized.

- The results of the statistical tests of the validation of the instrument are not properly expressed.

- The sentence: "Overall, these findings indicate support for the external validity..." is not methodologically correct. This is the internal validity of the instrument, not external validity (generalization of the results to the general population).

- The paragraph where the authors explain the preferred model is more appropriate in the discussion section. In the results section, the results are not discussed, only a statement of the results should be made.

- In the tables, all abbreviations should be specified in notes

Discussion

- The discussion must be worked on, providing a greater comparison with other studies and interpretation of results. They should avoid re-summarizing the results, putting paragraphs where not a single reference appears.

- The conclusion of the discussion section should not include interpretation of the results.
