# Supplementary material for: Confirmatory factor analysis and exploratory structural equation modelling of the factor structure of the Depression Anxiety and Stress Scales-21
Source: PLoS One. 2020 Jun 5;15(6):e0233998. doi: 10.1371/journal.pone.0233998 (PMC7274426; doi:10.1371/journal.pone.0233998)
Supplement: Attachment — Submitted filename: 2020 Response to Reviewers feedbak.docx [file pone.0233998.s003.docx]

04^th^ Feburary 2020

The Editor

PLOS ONE

Re: Revision of Manuscript PONE-D-19-32096
Confirmatory Factor Analysis and Exploratory Structural Equation Modeling of the Factor Structure of the Depression Anxiety Stress Scales-21

Dear Editor,

Thank you very much for arranging the review of the above mentioned paper that was submitted to PLOS ONE. We also like the reviewers for their valuable comments. These have been extremely useful for the revision of the paper. In the new revised paper we have considered these comments, and we believe that we have responded to all the revisions suggested. Our revisions are listed below, and are highlighted in yellow and green in the revised paper

**Responses to Reviewer #1:**

Overall, this was a robust and interesting evaluation of the measurement structure of the DASS-21. I have only minor comments for clarification.

Comment: The authors state several times that they are the first to test a bifactor ESEM of the DASS-21, but it should be noted that Johnson et al. (2016) also tested two Bifactor ESEM Models, including the BESEM Model tested in the present paper. See the following from the Methods section in Johnson et al. (2016):
Five established DASS-21 factor structures were fit first in CFA, and then in ESEM with ‘Target’ rotation:
1. 1-factor Model
2. 2-factor Model – Oblique model, two correlated factors representing physiological hyperarousal and generalised negativity, Duffy et al., (2005)
3. 3-factor Model – Oblique model, three correlated factors representing depression, anxiety, and stress, Lovibond and Lovibond (1995)
4. Bifactor Model A – Nested model, 3 independent factors representing depression, anxiety, and stress and a general negative affect factor, Henry and Crawford (2005)
5. Bifactor Model B – Nested model, 2 independent factors representing depression and stress and a general negative affect factor, Tully, Zajac, and Venning (2009)

Response. We have removed all instances where we have claimed (previously) that we are the first to test a bifactor ESEM of the DASS-21. Related to this, we have cited and discussed the work of Johnson et al. (2016), in particular their exploration and findings related to the bifactor ESEM model (p. 10, yellow highlight I n para 1).

Comment: A note on the wording: there is extensive reference to the method used as simply ‘ESEM’, but ESEM is a general framework for using EFA within structural models and does not indicate the type of EFA rotation used. The authors should note that the analyses used ‘Target’ rotation.

Response. As suggested we no longer refer to ESEM, but to ESEM (and also BESEM) with target rotation (highlighted in yellow throughout the paper).

Comment: The calculation of the Omega coefficient needs clarification. When using the WLSMV estimator, the Omega statistic should not be calculated in the same way as is done with the analysis of continuous items (Yang & Xia, 2019). A method for estimating coefficient omega for ordinal items has been proposed by Green and Yang (2009) and should be used instead.

Response. We have not made any change in this respect, for the reasons given in p. 15 (yellow highlight).

Comment: The authors should clarify the extent of missingness present in the data. WLSMV is a limited-information estimator and handles missingness using pairwise deletion. This approach may not be suitable for samples with large proportions of missingness.

Response. We covered this in p. 15 (yellow highlight).

Comment Minor correction: the estimator is not named ‘Weighted least square mean and variance adjusted chi-square’, just ‘Weighted least square mean and variance adjusted’, as both the standard errors and the chi-square statistic are adjusted.

Response. Corrected (p. 13, yellow highlight).

Comment: While the authors provide fit indices for each model, they do not provide the model comparison statistics (especially the Δχ2). While the authors mention that ‘The models did not differ from each other because the ΔCFI and ΔRMSEA values between all model pairs were less than 0.01, and 0.015 respectively.’, this is not the case. The ΔCFI was greater than 0.01 for the comparison between the CFA 3-F and all models except the BCFA 3-s-F.

Response. This mistake has been correct (p. 16, yellow highlight)

Comment: The authors state in the discussion ‘Consequently, it is possible that there may be another non-modeled higher order factor needed for the DASS-21.’ This is a simple hypothesis to test, as the authors could add a higher-order 3-factor model (i.e. the 3 factors as indicators of another latent variable).

Response. We now consider that based on our findings such an hypothesis is inappropriate, and have remove this statement. Thus the computation of the model suggested is irrelevant.

Comment: The authors mention several times that BESEM ‘provides the most advanced method to model the variances among the DASS-21 items…’. However, this title would likely go to Bayesian SEM, which can estimate both cross-loadings and residual covariances (whereas ESEM& BESEM can only estimate cross-loadings).

Response. We no longer refer to the BESEM approach as the most advanced method to model the variances among the DASS-21 items…’. Instead we say it is a “could be potentially a more sophisticated and arguably more advanced approach for studying the factor structure of the DASS-21” (p. 10, lines 11 & 12).

Comment: The authors’ conclusion that ‘…the total scores of the depression, anxiety, and stress scales could be used as measures of these constructs’ is incongruent with the findings of multiple cross-loadings in the ESEM models. Using sum scores for scale totals assumes that the scale is measured only by the items being summed, and that severity on these items is indicative only of severity on the construct of interest (measurement error aside). By identifying several cross-loading items, the authors have indicated that these assumptions do not hold, and that total scores may not be reliable.

Response. Given the suggestion by Reviewer 2 that the “conclusion of the discussion section should not include interpretation of the results” we have removed the paragraph that contains the information referred to by this reviewer. Thus this point in not made in the current version of the paper.

**Responses to Reviewer #2:**

The subject is of interest and the manuscript has been performed with quality methodological. However, some aspects should be worked on, namely the quality of the writing and the content to be expressed in each part of the manuscript.

Comment: Introduction

- The authors don't have provided literature to justify the need for testing the DASS-21. The beginning "The DASS-21 has been derived from...." is not an appropriate way to start a manuscript. It should include a brief introduction of the topic to be addressed, of the importance of this scale... And not start with a psychometric approach. In this same line, concepts such as CFA and ESSEM are first introduced to be explained in the final part of the introduction; this does not facilitate the understanding of the subject.

Response: Justification for the study is now provided (p. 10, green highlights). We begin the paper now with a a brief introduction of the topic to be addressed (p. 3 para 1, green highlights). We have revised the paper such that concepts such as CFA and ESSEM are not introduced first without explanation.

Comment: The authors make multiple statements throughout the translation that should be referenced, giving rise to confusion because they seem to have given it.

Response. Done throughout.

Comment: The introduction is excessively long and confusing in its presentation. It needs to be worked on in-depth. It is not properly structured, with ideas repeated throughout the text that do not facilitate understanding and a vision of progression and organization.

Response. We have reorganized the introduction, providing better structure, and removing repetitions.

Comment: Acronyms must be specified the first time they appear. Even the name of a scale.

Response. This has been conformed to.

Comment: On page 6, refer to "minor misspecifications", please clarify such general expressions.
Response. This has been clarified (p. 5, green highlight).

Method:
Comment - There is no need to indicate age by gender.
- Better express the statistical results (M = , SD = )
- The expression (t[736]=0.05, ns), is incorrectly expressed. Please include the statistic t correctly and the p.

Response. All the above have been revised (see “Participants” section, p. 11)

Comment: It is unnecessary to include the descriptive for the DASS items.

Response: Removed (see Table 1)

Comment: It would be convenient to include other types of sociodemographics in the table, such as the year, the gender, the nationality, socioeconomics level.

Response: As the information on age and gender have been provided in the text, we hve not provided this in the table. There is no information on nationality and socioeconomics level.

Comment: The last paragraph of the participants is for discussion, not methods.

Response: It still remain under participants, we fell that it provides information on the participants that is better at this point of the paper.

Comment: More detailed information needs to be provided on sample and recruitment, procedure.

Response: Additional information is now provided (p. 13, green highlight)

Comment: It would be recommended to reduce the analysis data section.

Response: We have done so.

Comment: It has not been included which type of statistical tests have been carried out in the validity of the tool.
Response: We have done so (p. 15, green highlight).

Results
Comment: Summarize the results in the text if they are set out in the tables.

Response: We have done so.

Comment: It is not necessary to explain in detail in the text the factorial loads of each of the items. Summarize and specify the different and/or relevant results. Also, organize it in a detailed way by the factor in each of the models. As it is now, it is confusing and disorganized.

Response: We have not made much changes in this respect. Our intention in the results section is to show how well the factors are defined by the individual items, and latter to discuss which items are problematic. Dscussion of these will make little sense if details of individual items are not covered in the results section.

Comment: The results of the statistical tests of the validation of the instrument are not properly expressed.

Response: We have rewritten this section (pp. 20 & 21, green highlight).

Comment: The sentence: "Overall, these findings indicate support for the external validity..." is not methodologically correct. This is the internal validity of the instrument, not external validity (generalization of the results to the general population).

Response. We think that examining the relations of the DASs-21 factors with IA and HI test the external (and not internal) validity of the DASS-21 factors.

- The paragraph where the authors explain the preferred model is more appropriate in the discussion section. In the results section, the results are not discussed, only a statement of the results should be made.

Response: This is now only in the discussion.

Comment: In the tables, all abbreviations should be specified in notes
Response: Done

Discussion

Comment: The discussion must be worked on, providing a greater comparison with other studies and interpretation of results. They should avoid re-summarizing the results, putting paragraphs where not a single reference appears.

Response: In our discussion, we have providing a greater comparison with other studies and made interpretation of results. Although there are paragraphs without reference, we consider these paragraphs as essential to explaining our results.

Comment: The conclusion of the discussion section should not include interpretation of the results.

Response: the interpretation of results has been removed.

As you will notice we have addressed all the comments and suggestions made by the reviewer. We hope that the changes meet with your satisfaction and we look forward to hearing from you soon.

Thank you.

Yours sincerely,

Authors
