# Supplementary material for: Confirmatory factor analysis and exploratory structural equation modelling of the factor structure of the Depression Anxiety and Stress Scales-21
Source: PLoS One. 2020 Jun 5;15(6):e0233998. doi: 10.1371/journal.pone.0233998 (PMC7274426; doi:10.1371/journal.pone.0233998)
Supplement: Attachment — Submitted filename: 2020 Response 2 to Reviewers feedbak.docx [file pone.0233998.s004.docx]

15^h^ May 2020

The Editor

PLOS ONE

Re: Revision of Manuscript PONE-D-19-32096
Confirmatory Factor Analysis and Exploratory Structural Equation Modeling of the Factor Structure of the Depression Anxiety Stress Scales-21

Dear Editor,

Thank you very much for arranging the second review of the above mentioned paper that was submitted to PLOS ONE. We also like the reviewer 2 (who did not request any additional modifications) and reviewer 1 for recommending the calculation of the categorical omega via the R-MBESS ci-reliability function. In the new revised paper we have considered this comment raised by reviewer 1. We believe that we have now responded to this final revision suggested. The revision(s) is listed below, and is highlighted in yellow in this second revised version of the paper.

**Responses to Reviewer #1:**

Comment: In their revision, the authors stated that:

"It may be worth noting that Yang and Green (2015) have argued that when

using the WLSMV estimator, the omega should not be calculated in the same way as is done

with the analysis of continuous items (Yang & Xia, 2019). This is especially so if the sample size

is less than 500 as omega values would be underestimated. Instead, Bayesian estimation methods

have been proposed (Yang & Xia, 2019). However, as our sample size was well above 500 (N =

738), we used the usual method applied to continuous items for computing omega values."

However, these are not the conclusions of the studies that they are citing. The referenced studies show that *categorical* Omega (i.e. the correct treatment of omega with WLSMV estimation) can be biased in small sample sizes, not that the standard (continuous) version is biased. In fact, the referenced studies do not predicate the appropriateness of the continuous method on sample size at all:

"However, x and factor scores have different metrics and their relationship is no longer linear. Describing the relation of the observed scores to the true scores using the Pearson correlation (or the square of the correlation) is thus inappropriate (Lord & Novick, 1968). Consequently, Definition 2 and Definition 3 are not appropriate for defining a reliability coefficient for the scale scores." (Yang & Xia, 2018, p. 21).

The authors need to use categorical Omega here, otherwise the assumptions on which they estimate reliability (continuous observations) are different from the assumptions used to estimate the models (ordinal observations). If unsure, the simplest way to achieve this is using the ci.reliability() function in the MBESS R package.

Response. Thank you for your comment. The relevant part of the text has now been modified. Furthermore, we have computed and reported categorical Omega using the the ci.reliability function in the MBESS R package and perc interval type (see p.17, yellow highlight; and p. 22, yellow highlight). As we have been using M-Plus explicitly until now, your review point has provided us the opportunity to get more familiar with R and we are honestly grateful to you for that.

**Responses to Reviewer #2:**

Comment: No additional comments.

Response: Thank you for the time you devoted in the consideration of our manuscript and the effort you made to help us improve it.

As you will notice we have addressed all the comments and suggestions made by the reviewer. We hope that the changes meet with your satisfaction and we look forward to hearing from you soon.

Thank you.

Yours sincerely,

Authors
